# Subclinical vascular inflammation in subjects with normal weight obesity and its association with body Fat: an ^18^ F-FDG-PET/CT study

**DOI:** 10.1186/1475-2840-13-70

**Published:** 2014-04-04

**Authors:** Shinae Kang, Chanhee Kyung, Jong Suk Park, Sohee Kim, Seung-Pyo Lee, Min Kyung Kim, Hye Kyung Kim, Kyung Rae Kim, Tae Joo Jeon, Chul Woo Ahn

**Affiliations:** 1 Department of Internal Medicine, Gangnam Severance Hospital, Yonsei University College of Medicine, 211 Eonju-ro, Gangnam-gu, Seoul, Korea; 2 Severance Institute for Vascular and Metabolic Research, Yonsei University College of Medicine, 211 Eonju-ro, Gangnam-gu, Seoul, Korea; 3 Cardiovascular Center and Department of Internal Medicine, Seoul National University Hospital, 101 Daehak-ro, Jongro-gu, Seoul, Korea; 4 Department of Family Medicine, Health Promotion Center, Gangnam Severance Hospital, Yonsei University College of Medicine, 211 Eonju-ro, Gangnam-gu, Seoul, Korea; 5 Department of Nuclear Medicine, Gangnam Severance Hospital, Yonsei University College of Medicine, 211 Eonju-ro, Gangnam-gu, Seoul, Korea

**Keywords:** Normal weight obesity, Fat, Atherosclerosis, ^18^ F-FDG-PET/CT, Vascular inflammation

## Abstract

**Background:**

Although body mass index (BMI) is the most widely accepted parameter for defining obesity, recent studies have indicated a unique set of patients who exhibit normal BMI and excess body fat (BF), which is termed as normal weight obesity (NWO). Increased BF is an established risk factor for atherosclerosis. However, it is unclear whether NWO subjects already have a higher degree of vascular inflammation compared to normal weight lean (NWL) subjects; moreover, the association of BF with vascular inflammation in normal weight subjects is largely unknown.

**Methods:**

NWO and NWL subjects (n = 82 in each group) without any history of significant vascular disease were identified from a 3-year database of consecutively recruited patients undergoing ^18^ F-fluorodeoxyglucose positron emission tomography/computed tomography (^18^ F-FDG-PET/CT) at a self-referred Healthcare Promotion Program. The degree of subclinical vascular inflammation was evaluated using the mean and maximum target-to-background ratios (TBRmean and TBRmax) of the carotid artery, which were measured by ^18^ F-FDG-PET/CT (a noninvasive tool for assessing vascular inflammation).

**Results:**

We found that metabolically dysregulation was greater in NWO subjects than in NWL subjects, with a significantly higher blood pressure, higher fasting glucose level, and worse lipid profile. Moreover, NWO subjects exhibited higher TBR than NWL subjects (TBRmean: 1.33 ± 0.16 versus 1.45 ± 0.19, p < 0.001; TBRmax: 1.52 ± 0.23 versus 1.67 ± 0.25, p < 0.001). TBR was significantly associated with total BF (TBRmean: r = 0.267, p = 0.001; TBRmax: r = 0.289, p < 0.001), age (TBRmean: r = 0.170, p = 0.029; TBRmax: r = 0.165, p = 0.035), BMI (TBRmean: r = 0.184, p = 0.018; TBRmax: r = 0.206, p = 0.008), and fasting glucose level (TBRmean: r = 0.157, p = 0.044; TBRmax: r = 0.182, p = 0.020). In multiple linear regression analysis, BF was an independent determinant of TBRmean and TBRmax, after adjusting for age, BMI, and fasting glucose level (TBRmean: regression coefficient = 0.020, p = 0.008; TBRmax: regression coefficient = 0.028, p = 0.005). Compared to NWL, NWO was also independently associated with elevated TBRmax values, after adjusting for confounding factors (odds ratio = 2.887, 95% confidence interval 1.206–6.914, p = 0.017).

**Conclusions:**

NWO is associated with a higher degree of subclinical vascular inflammation, of which BF is a major contributing factor. These results warrant investigations for subclinical atherosclerosis in NWO patients.

## Background

According to National Health and Nutrition Examination Survey (NHANES) and Korean National Health and Nutrition Examination Survey (KNHANES) data, the prevalence of obesity has increased to 30% in USA [1] and 30.9% in Korea [2]. Obesity is a well-known cause of cardiovascular disease (CVD) and all-cause mortality [3-5]. Classically, obesity has been defined as an excess of body weight compared to the height, which has been simplified as a single value, termed as the body mass index (BMI) [6].

The limitation of BMI is that it can misclassify subjects who have lower lean mass but higher fat mass as non-obese [7]. This inherent limitation of BMI in the accurate diagnosis of obesity is observed to a greater extent among Asians and the elderly, who commonly have lower lean body mass [8-10]. Consequently, the new concept of normal weight obesity (NWO) had been proposed. NWO subjects have normal BMI and an elevated body fat percentage (BF%) [11], and may be at a high risk for cardiometabolic disease [12-16]. Considering that fat itself is a major contributing factor on CVD in obese subjects [5], methods to estimate the risk of CVD associated with BF% or absolute fat mass can be accurate and helpful for assessing early cardiometabolic disease risk in the NWO population.

Inflammation is a major driving factor of atherosclerosis progression [17] and a major determinant of plaque rupture [18], the results of which may be devastating. Therefore, accurate assessment of vascular inflammation rather than the extent of luminal stenosis may be more essential for preventing CVD [19]. The uptake of ^18^ F-fluorodeoxyglucose (^18^ F-FDG) in the vessel correlates very well with the degree of macrophage infiltration [20], because the macrophages preferentially utilize glucose, particularly under anaerobic conditions [21,22]. Based on these findings, the usefulness of ^18^ F-FDG positron emission tomography/computed tomography (^18^ F-FDG-PET/CT) has been demonstrated when evaluating vascular inflammation [23-26]. Therefore, in the present study, we aimed to determine the degree of subclinical vascular inflammation in NWO subjects and the contributing factors associated with the inflammation. Hence, we used a large database consecutively recruited from the patients undergoing ^18^ F-FDG-PET/CT from a self-referred healthcare promotion program to identify patients without any history of clinical cerebrovascular disease.

## Methods

### Study design and population

This cross-sectional study was designed to evaluate the effect of BF on vascular inflammation of the carotid arteries in subjects with a normal BMI. Asymptomatic subjects self-referred for ^18^ F-FDG-PET/CT examination from March 2010 to February 2013 in a health checkup program at the Health Promotion Center, Gangnam Severance Hospital, were consecutively registered (N = 1003; Men: n = 602; Women: n = 401). All subjects underwent a medical examination, including clinical history, physical examination, blood analysis, abdominal ultrasound, and ^18^ F-FDG-PET/CT.

**Figure 1:**
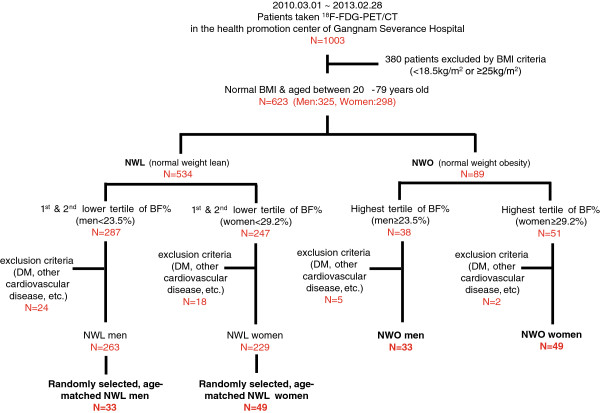
**Schematic diagram and the process of the registration of subjects. **^18^ F-FDG-PET/CT, ^18^ F-fludeoxyglucose positron emission tomography/computed tomography; BMI, body mass index; NWL, normal weight lean; NWO, normal weight obesity; BF, body fat; DM, diabetes mellitus.

We divided the whole population by gender-specific BF% tertiles and defined NWO subjects as those with a normal BMI (18.5–24.9 kg/m^2^) and the highest tertile of BF% (Men, ≥23.5%, Women, ≥29.2%) in line with a previous study^15^, thus yielding 89 NWO subjects (Men: N = 38; Women: N = 51). Subjects who were previously diagnosed with diabetes mellitus and/or taking anti-diabetic medication were excluded. Moreover, subjects with previously diagnosed malignancy within 5 years of PET/CT examination, chronic inflammatory disease, or other previous disease of the carotid artery were excluded. Finally, 82 NWO subjects were included in the present study. Moreover, 82 control normal weight lean (NWL) subjects (Men, <23.5%; Women, <29.2%)—i.e., normal BMI subjects who were in the lower two tertiles for gender-specific BF%—were randomly selected from sex-matched subjects using random numbers uniformly distributed between 0 and 1 (Figure 1). In addition, we constructed another database comprising 63 subjects each in the NWL and NWO groups who were matched for sex and BMI values using greedy matching techniques. The exclusion criteria of the NWL subjects were identical to those for the NWO subjects. The protocol of this study was approved by the institutional review board of Gangnam Severance Hospital, and all subjects provided written informed consent for the ^18^ F-FDG-PET/CT examination.

### Questionnaire

The current and past medical history of disease, medication, presence of CVD risk factors (smoking, alcohol, and family history of premature CVD), and other information on medico-social history were self-reported via a routine questionnaire before the health checkup.

### Anthropometric measurement

All subjects were requested to fast for more than 8 hours before all biochemical and anthropometric examinations. Body weight was measured by electronic load to the nearest 0.01 kg, with the parents wearing a light gown without shoes for the health examination. Height was measured to the nearest 0.1 cm. BMI was calculated as the weight in kilograms divided by the square of height in meters (kg/m^2^). The BF%, total body fat, visceral fat, subcutaneous fat, and muscle mass were measured using the bioelectrical impedance (BI) body composition analyzer X-scan plusII (Jawon Medical, Seoul, Korea) [27]. In brief, the tetra-polar electrode method with 8 touch electrodes placed on both hands and feet facilitated the estimation of the BI value in the abdominal area, which was evaluated by the current intercrossing between each hand and foot. By using the waist-to-hip ratio, body weight, height, age, sex, and the BI value of the abdominal area, the Jawon Medical Company created a formula specific for Korean individuals to calculate the visceral fat amount. The subcutaneous fat amount was estimated by excluding the visceral fat from the total fat amount [28,29]. Systolic and diastolic blood pressure was measured using BP203RV II (Colin Corp., Aichi, Japan) after resting for more than 15 min in the sitting position. The presence of fatty liver was assessed using a Philips IU-22 Ultrasound system (Philips Healthcare, Andover, MA) by an expert radiologist.

### Biochemical measurement

Glucose was measured by the glucose oxidase method using a 747 Automatic Analyzer (Hitachi, Tokyo, Japan). Glycated hemoglobin A1c (HbA1c) level was measured by high-performance liquid chromatography (Cobas Integra 800, Roche, Mannheim, Germany). The levels of total cholesterol, high-density lipoprotein cholesterol (HDL-cholesterol), triglyceride, C-reactive protein (CRP), aspartate aminotransferase (AST), and alanine aminotransferase (ALT) were measured by an enzymatic colorimetric method (Daiichi, Hitachi). Low-density lipoprotein cholesterol (LDL-cholesterol) content was calculated according to the Friedewald formula.

### ^18^ F-FDG-PET/CT imaging and image analysis

All patients fasted at least 8 h before the injection of ^18^ F-FDG, after ensuring that serum glucose levels at this time point did not exceed 150 mg/dL. The injection dose of ^18^ F-FDG was 5.18 MBq/kg (0.14 mCi/kg). ^18^ F-FDG-PET images were obtained using a standard PET/CT scanner (Biograph TruePoint 40, Siemens Healthcare, Erlangen, Germany) and all data were acquired in the 3D mode with a 1.5-min scan time per bed. Tomographic PET images were reconstructed by using an iterative method involving ordered subset expectation maximization, under the condition of 2 iterations and 21 subsets. The matrix of reconstructed PET images using this method was 128 × 128, and the slice thickness was 5 mm. Low-dose CT scans for attenuation correction and identification of anatomical landmarks were obtained immediately before the PET scan.

Image analysis was conducted using a dedicated PET/CT workstation (Syngo 2009A, Siemens Healthcare) according to previous reports [20,24-26]. In detail, for exact measurement of the carotid artery and jugular vein, the overall diameter, course, and variation of cervical vasculature were first reviewed using CT images, after which the metabolic activity of the artery and vein was estimated by using PET/CT fusion images prior to region of interest (ROI) measurements. A circular ROI was used in most slices of the carotid arteries, whereas an oval or circular ROI was used for the jugular vein depending on its contour. Each ROI was intended to include the entire wall of the artery and vein. The measurement of the uptake value (SUV) was started from the highest level of the common carotid artery before bifurcation to the external and internal carotid branches (mostly around the level of the hyoid bone), and 10 ROIs were consecutively measured from the inferior level up to the origin of the carotid artery from the aortic arch. Eight ROIs were used to measure the jugular vein SUV, and their locations were similar to those of the carotid artery. The maximal (SUVmax) and mean SUV (SUVmean) were measured in the carotid artery and the jugular vein, respectively, after which the target-to-background ratio (TBR) was calculated using the following equations: Mean TBR (TBRmean) = (mean SUV of carotid artery)/(mean SUV of jugular vein); Maximum TBR (TBRmax) = (maximum SUV of carotid artery)/(mean SUV of jugular vein) [20,24-26].

### Statistical analyses

Anthropometric, clinical, and biochemical data are presented as the mean ± standard deviation (SD) for continuous variables and absolute numbers (percentage) for categorical variables. An independent two-sample *t*-test or paired sample *t*-test was used to evaluate the differences in the continuous variables between NWL and NWO subjects after testing each variable for normality with the Shapiro-Wilk test. The χ^2^ test or Fisher’s exact test was used to compare categorical variables between the NWL and NWO groups, as appropriate. The degree of a relationship between putative CVD risk factors and TBRmean or TBRmax was expressed by Pearson’s correlation coefficient (r). Multiple stepwise linear regression analysis was used to determine the independent predictors of TBRmean and TBRmax. Logistic regression models were analyzed to evaluate the odds ratio (OR) for TBRmax elevation in the NWO group compared to the NWL group. All statistical analyses were performed using adequate software (SPSS version 20.0, Chicago, IL). A p value of <0.05 was considered to be statistically significant.

## Results

### Clinical and biochemical characteristics of the study population

The mean age of the subjects was 54 years, and 18% of the subjects had hypertension. The NWO subjects had a higher BMI, larger waist-to-hip ratio, and greater amount of visceral and subcutaneous fat. There was no difference in total muscle mass between the groups. NWO subjects had significantly higher systolic and diastolic BP than NWL subjects. Although there was no difference in HbA1c levels, the fasting plasma glucose level was significantly higher in the NWO group. In addition, NWO subjects had higher serum triglyceride and lower HDL-cholesterol levels. No differences in current anti-hypertensive drug, statin, and anti-platelet use were noted between the groups. Detailed comparisons of each parameter are summarized in Table 1. Moreover, we analyzed these clinical and biochemical parameters after dividing the study population based on gender. The difference in the metabolic profile tended to be significant between the female subjects in the NWO and NWL groups, whereas certain parameters such as blood pressure and CRP were not significantly different in the male subjects between the NWO and NWL groups (Table 2).

**Table 1:** Clinical and biochemical characteristics of the study subjects

	**Total (N = 164)**	**NWL (N = 82)**	**NWO (N = 82)**	**p-value**
Age (years)	54.1 ± 9.4	52.9 ± 9.3	55.3 ± 9.4	0.101
Gender (N, (%))	66 (40)	33 (40)	33 (40)	>0.999
Body mass index (kg/m^2^)	22.7 ± 1.7	21.7 ± 1.6	23.8 ± 0.9	<0.001
Waist to hip ratio	0.87 ± 0.08	0.84 ± 0.08	0.89 ± 0.07	<0.001
Body fat (%)	27.1 ± 5.0	24.1 ± 4.5	30.1 ± 3.3	<0.001
Body fat (kg)	16.2 ± 3.0	14.0 ± 2.6	18.4 ± 1.7	<0.001
Visceral fat (kg)	2.0 ± 0.6	1.6 ± 0.4	2.5 ± 0.4	<0.001
Subcutaneous fat (kg)	14.1 ± 2.7	12.3 ± 2.3	15.9 ± 1.6	<0.001
Muscle mass (kg)	40.5 ± 6.5	41.2 ± 6.9	39.7 ± 6.1	0.147
Systolic BP (mmHg)	121.9 ± 14.9	118.7 ± 14.7	125.1 ± 14.4	0.005
Diastolic BP (mmHg)	76.3 ± 9.4	74.7 ± 9.4	78.0 ± 9.1	0.022
Fasting plasma glucose (mmol/L)	5.24 ± 0.66	4.98 ± 0.46	5.51 ± 0.72	<0.001
HbA1c (%)	5.6 ± 0.5	5.5 ± 0.4	5.7 ± 0.6	0.094
Total cholesterol (mmol/L)	5.12 ± 0.89	5.11 ± 0.90	5.13 ± 0.89	0.859
Triglyceride (mmol/L)	1.14 ± 0.57	1.01 ± 0.51	1.27 ± 0.61	0.003
HDL-cholesterol (mmol/L)	1.37 ± 0.32	1.46 ± 0.35	1.29 ± 0.261	0.001
LDL-cholesterol (mmol/L)	3.14 ± 0.80	3.09 ± 0.80	3.19 ± 0.80	0.397
CRP (mg/L)	1.6 ± 3.1	1.2 ± 3.2	2.0 ± 2.9	0.096
AST (IU/L)	22.4 ± 8.8	22.3 ± 10.6	22.4 ± 6.7	0.916
ALT (IU/L)	22.9 ± 14.5	20.7 ± 11.6	25.2 ± 16.7	0.049
Fatty Liver (N, (%))	53 (32.3)	14 (17.1)	39 (47.6)	<0.001
Antihypertension drug (N, (%))	24 (17.1)	10 (12.2)	14 (17.1)	0.377
Statin (N, (%))	14 (8.6)	8 (9.8)	6 (7.3)	0.576
Antiplatelet agent (N, (%))	15 (9.0)	7 (8.5)	8 (9.8)	0.786

NWL, normal weight lean; NWO, normal weight obesity; N, number; BP, blood pressure; HbA1c, glycated hemoglobin A1c; HDL, high density lipoprotein; LDL, low density lipoprotein; AST/ALT, aspartate/alanine aminotransferase; CRP, C-reactive protein. Data are presented as mean ± SD or number (percentages). The p values represent differences between groups determined by an independent two-sample *t*-test for continuous variables and the χ^2^ test or the Fisher’s exact test for categorical variables.

**Table 2:** Clinical and biochemical characteristics of the study subjects by gender

	**Men**			***p***	**Women**			***p***
	**Total (N = 66)**	**NWL (N = 33)**	**NWO (N = 33)**		**Total (N = 98)**	**NWL (N = 49)**	**NWO (N = 49)**	
Age (years)	56.0 ± 9.1	55.5 ± 8.8	56.4 ± 9.6	0.699	52.9 ± 9.5	51.2 ± 9.3	54.6 ± 9.4	0.071
Body mass index (kg/m2)	23.3 ± 1.3	22.4 ± 1.3	24.2 ± 0.6	<0.001	22.3 ± 1.8	21.1 ± 1.5	23.5 ± 1.0	<0.001
Waist to hip ratio	0.94 ± 0.04	0.91 ± 0.04	0.97 ± 0. 02	<0.001	0.82 ± 0.05	0.79 ± 0.05	0.84 ± 0.02	<0.001
Body fat (%)	23.4 ± 4.1	20.2 ± 3.6	26.5 ± 1.0	<0.001	29.6 ± 3.8	26.6 ± 2.9	32.6 ± 1.4	<0.001
Body fat (kg)	15.5 ± 3.2	13.2 ± 2.6	17.9 ± 1.5	<0.001	16.6 ± 3.0	14.5 ± 2.5	18.7 ± 1.7	<0.001
Visceral fat (kg)	2.3 ± 0.6	1.8 ± 0.5	2.8 ± 0.2	<0.001	1.8 ± 0.5	1.4 ± 0.4	2.3 ± 0.3	<0.001
Subcutaneous fat (kg)	13.1 ± 2.6	11.3 ± 2.2	15.0 ± 1.3	<0.001	14.7 ± 2.6	13.0 ± 2.2	16.5 ± 1.5	<0.001
Muscle mass (kg)	47.0 ± 4.6	48.1 ± 4.7	45.9 ± 4.2	0.047	36.1 ± 3.1	36.6 ± 3.4	35.6 ± 2.8	0.122
Systolic BP (mmHg)	125.0 ± 13.7	122.5 ± 13.6	127.6 ± 13.4	0.134	119.8 ± 15.3	116.1 ± 14.9	123.5 ± 15.0	0.017
Diastolic BP (mmHg)	78.9 ± 8.2	77.9 ± 8.3	79.9 ± 8.2	0.319	74.6 ± 9.7	72.5 ± 9.6	76.7 ± 9.5	0.031
Fasting plasma glucose (mmol/L)	5.43 ± 0.66	5.09 ± 0.48	5.76 ± 0.66	<0.001	5.12 ± 0.62	4.90 ± 0.42	5.33 ± 0.71	<0.001
HbA1c (%)	5.7 ± 0.5	5.6 ± 0.4	5.8 ± 0.6	0.245	5.6 ± 0.5	5.5 ± 0.5	5.6 ± 0.5	0.213
Total cholesterol (mmol/L)	5.03 ± 0.95	5.14 ± 1.01	4.93 ± 0.91	0.364	5.19 ± 0.95	5.10 ± 0.84	5.29 ± 0.86	0.283
Triglyceride (mmol/L)	1.36 ± 0.71	0.19 ± 0.68	1.53 ± 0.71	0.048	1.36 ± 0.71	1.19 ± 0.68	1.53 ± 0.71	0.010
HDL-cholesterol (mmol/L)	1.22 ± 0.26	1.31 ± 0.29	1.12 ± 0.20	0.004	1.48 ± 0.31	1.56 ± 0.35	1.41 ± 0.24	0.016
LDL-cholesterol (mmol/L)	3.14 ± 0.85	3.18 ± 0. 89	3.11 ± 0.83	0.756	3.15 ± 0.77	0.04 ± 0.75	3.26 ± 0.79	0.155
CRP (mg/L)	2.4 ± 4.50	1.8 ± 5.1	2.9 ± 4.0	0.322	1.1 ± 1.3	0.8 ± 0.7	1.4 ± 1.6	0.026
AST (IU/L)	24.9 ± 11.6	25.7 ± 14.7	24.1 ± 7.5	0.593	20.6 ± 5.7	20.0 ± 5.6	21.3 ± 5.9	0.271
ALT (IU/L)	27.9 ± 15.0	24.7 ± 12.2	31.1 ± 16.9	0.083	19.6 ± 13.2	18.0 ± 10.5	21.2 ± 15.4	0.239
Fatty Liver (N,%)	29 (44.0)	7 (21.2)	22 (66.7)	<0.001	24 (24.5)	7 (14.7)	17 (34.7)	0.019
Antihypertension drug (N, (%))	15 (22.7)	6 (18.2)	9 (27.3)	0.378	9 (9.2)	4 (8.1)	5 (10.2)	>0.999
Statin (N,%)	7 (10.6)	2 (6.1)	5 (15.2)	0.427	7 (7.1)	6 (12.2)	1 (2.0)	0.111
Antiplatelet agent (N,%)	7 (10.6)	1 (21.2)	6 (12.1)	0.105	8 (8.1)	6 (12.2)	2 (4.1)	0.268

NWL, normal weight lean; NWO, normal weight obesity; N, number; BP, blood pressure; HbA1c, glycated hemoglobin A1c; HDL, high density lipoprotein; LDL, low density lipoprotein; AST/ALT, aspartate/alanine aminotransferase; CRP, C-reactive protein. Data are presented as mean ± SD or number (percentages). The p values represent differences between groups determined by an independent two-sample *t*-test for continuous variables and the χ^2^ test or the Fisher’s exact test for categorical variables.

### Evaluation of vascular inflammation by ^18^ F-FDG-PET/CT between NWL and NWO subjects

**Figure 2:**
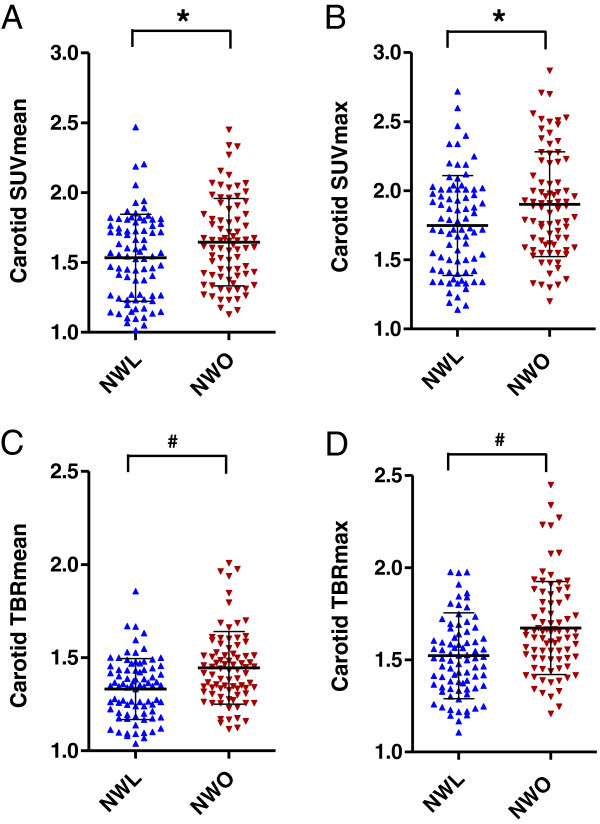
**Scatter plot and comparison of the **^**18**^ **F-FDG-PET/CT results between NWL and NWO subjects.** A scatter plot of the ^18^ F-FDG-PET/CT results was drawn, and a comparison between the NWL and NWO subjects was performed. **(A)** Carotid mean SUV, **(B)** carotid maximum SUV, **(C)** carotid mean TBR, and **(D)** carotid maximum TBR. ^18^ F-FDG-PET/CT, ^18^ F-fludeoxyglucose positron emission tomography/computed tomography; SUV, standardized uptake value; TBR, target-to-background ratio; NWL, normal weight lean; NWO, normal weight obesity. Horizontal lines in the graph indicate means, whereas perpendicular lines denote the standard deviation. ^*^p < 0.05 versus NWL. ^#^p < 0.001 versus NWL.

**Figure 3:**
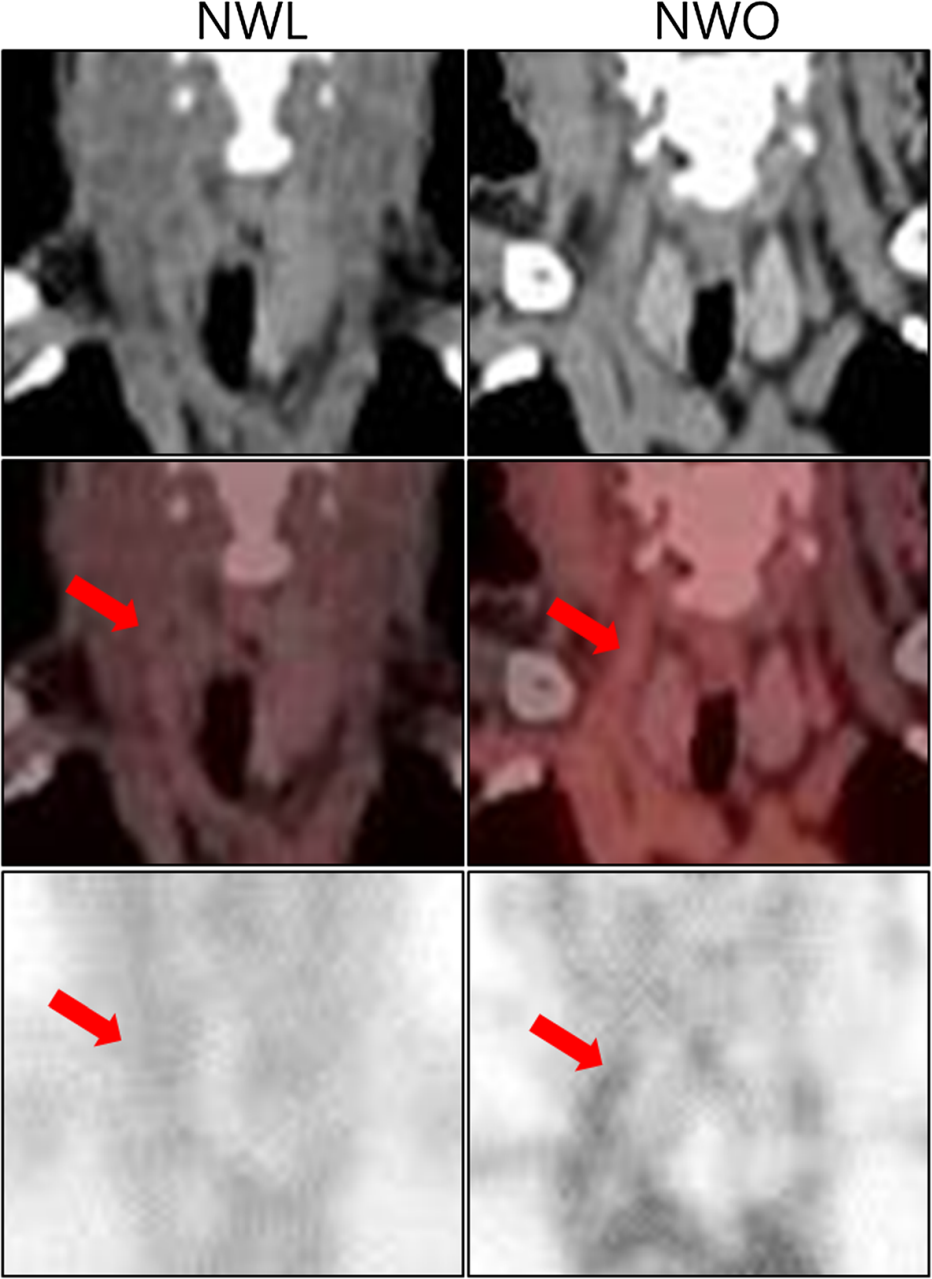
**Representative figures indicating the SUV uptake at the carotid artery in NWL and NWO subjects.** Coronal image of ^18^ F-FDG-PET/CT around the carotid artery is presented as an unenhanced CT image, a fusion image of CT and PET, and a maximum intensity projection (MIP) image, from the upper panel to the lower panel. The red arrow indicates the carotid artery. ^18^ F-FDG-PET/CT, ^18^ F-fludeoxyglucose positron emission tomography/computed tomography; SUV, standardized uptake value; NWL, normal weight lean; NWO, normal weight obesity.

Compared to the NWL group, the NWO group had a significantly higher SUVmean and SUVmax at the carotid artery (SUVmean: 1.53 ± 0.31 versus 1.64 ± 0.31, p = 0.024; SUVmean: 1.75 ± 0.36 versus 1.90 ± 0.38, p = 0.009) (Table 3, Figures 2A and B, Figure 3). However, there was no difference in the SUVmean at the jugular vein between the two groups. TBRmean and TBRmax—the corrected values of carotid SUVmean and SUVmax divided by the SUVmean of the jugular vein in each individual, respectively—were also higher in the NWO population (TBRmean: 1.33 ± 0.16 versus 1.45 ± 0.19, p < 0.001; TBRmax: 1.52 ± 0.23 versus 1.67 ± 0.25, p < 0.001) (Table 3, Figures 2C and D). As the degree of vascular inflammation may be affected by the coexistence of hypertension or statin use, we also analyzed the degree of carotid inflammation after excluding subjects with these factors. However, the difference was consistently significant even in patients without anti-hypertensive or statin use (TBRmean: 1.33 ± 0.16 versus 1.44 ± 0.21, p < 0.001; TBRmax: 1.52 ± 0.24 versus 1.67 ± 0.03, p < 0.001) (NWL: n = 67; NWO: n = 64). We also analyzed the difference in vascular inflammation between NWL and NWO subjects from the total population who were equally matched by sex and BMI. We observed a significant difference in the degree of carotid inflammation after matching for BMI (TBRmean: 1.24 ± 0.25 versus 1.44 ± 0.21, p < 0.001; TBRmax: 1.42 ± 0.29 versus 1.68 ± 0.32, p < 0.001) (n = 63 in each group). Moreover, we analyzed the vascular inflammation according to sex. We observed a significant difference in vascular inflammation in male subjects between the NWL and NWO groups (TBRmean: 1.33 ± 0.15 versus 1.47 ± 0.16, p = 0.001; TBRmax: 1.50 ± 0.18 versus 1.71 ± 0.22, p < 0.001) and in female subjects between the NWL and NWO groups (TBRmean: 1.33 ± 0.20 versus 1.43 ± 0.22, p = 0.015; TBRmax: 1.54 ± 0.26 versus 1.65 ± 0.27, p = 0.038) (Table 4).

**Table 3:** **Comparison of **^**18**^ **F-FDG-PET/CT results between the NWL and NWO subjects**

	**Total (N = 164)**	**NWL (N = 82)**	**NWO (N = 82)**	**p-value**
SUVmean, jugular vein	1.15 ± 0.22	1.16 ± 0.23	1.15 ± 0.22	0.745
SUVmean, carotid artery	1.59 ± 0.32	1.53 ± 0.31	1.64 ± 0.31	0.024
SUVmax, carotid artery	1.83 ± 0.38	1.75 ± 0.36	1.90 ± 0.38	0.009
TBRmean, carotid artery	1.39 ± 0.19	1.33 ± 0.16	1.45 ± 0.19	<0.001
TBRmax, carotid artery	1.60 ± 0.25	1.52 ± 0.23	1.67 ± 0.25	<0.001

NWL, normal weight lean; NWO, normal weight obesity; N, number; SUVmean/max, mean/maximum standardized uptake value; TBRmean/max, mean/maximum target-to-background ratio. Data are presented as mean ± SD. The p values represent differences between groups determined by the independent two-sample *t*-test.

**Table 4:** **Comparison of **^**18**^ **F-FDG-PET/CT results between the NWL and NWO subjects by gender**

	**Men**			***p***	**Women**			***P***
	**Total**	**NWL**	**NWO**		**Total**	**NWL**	**NWO**	
	**(N = 66)**	**(N = 33)**	**(N = 33)**		**(N = 98)**	**(N = 49)**	**(N = 49)**	
SUVmean, jugular vein	1.15 ± 0.19	1.18 ± 1.19	1.12 ± 0.19	0.267	1.15 ± 0.24	1.15 ± 0.25	1.16 ± 0.24	0.736
SUVmean, carotid artery	1.60 ± 0.26	1.56 ± 0.28	1.63 ± 0.25	0.232	1.59 ± 0.35	1.52 ± 0.33	1.65 ± 0.35	0.058
SUVmax, carotid artery	1.83 ± 0.33	1.76 ± 0.31	1.90 ± 0.33	0.067	1.82 ± 0.41	1.74 ± 0.40	1.90 ± 0.41	0.057
TBRmean, carotid artery	1.40 ± 0.17	1.33 ± 0.15	1.47 ± 0.16	0.001	1.38 ± 0.22	1.33 ± 0.20	1.43 ± 0.22	0.015
TBRmax, carotid artery	1.60 ± 0.23	1.50 ± 0.18	1.71 ± 0.22	<0.001	1.59 ± 0.27	1.54 ± 0.26	1.65 ± 0.27	0.038

NWL, normal weight lean; NWO, normal weight obesity; N, number; SUVmean/max, mean/maximum standardized uptake value; TBRmean/max, mean/maximum target-to-background ratio. Data are presented as mean ± SD. The p values represent differences between groups determined by the independent two-sample *t*-test.

### Correlation between the degree of vascular inflammation and various CVD risk factors

**Figure 4:**
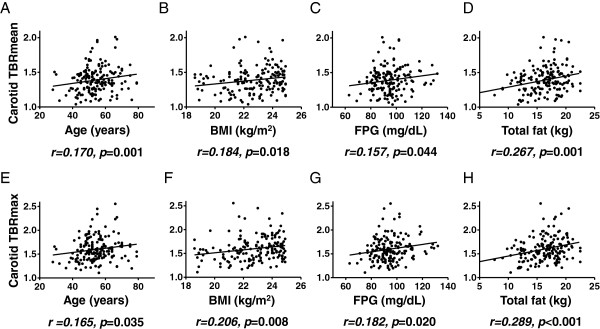
**Correlation between carotid TBR, various clinical factors, and body fat mass.** A correlation graph was drawn between the carotid **(A-D)** mean or **(E-H)** maximum TBR and age, BMI, fasting plasma glucose levels, and total body fat. The degree of relationship between putative CVD risk factors and TBRmean or TBRmax was expressed using Pearson’s correlation coefficient (r). TBR, target-to-background ratio; BMI, body mass index; FPG, fasting plasma glucose. p < 0.05 was regarded as statistically significant.

The correlation between vascular inflammation and various CVD risk factors was analyzed. Age, BMI, and the fasting plasma glucose level were significantly associated with both TBRmean and TBRmax (Table 5 and Figures 4A-C, 4E-G). We also analyzed the correlation between vascular inflammation and fat mass. Total, visceral, and subcutaneous fat mass were all significantly correlated with the degree of vascular inflammation; this degree of correlation was better than that observed for the traditional CVD risk factors of age, BMI, and fasting plasma glucose level (Table 5 and Figure 4D and H). However, none of the other anthropometric or biochemical characteristics correlated with TBRmean or TBRmax.

**Table 5:** **Correlation between the degree of TBR by **^**18**^ **F-FDG-PET/CT and various CVD risk factors**

	**TBRmean**		**TBRmax**	
	**r**	**p-value**	**r**	**p-value**
Age	0.170	0.029	0.165	0.035
Body mass index	0.184	0.018	0.206	0.008
Waist to hip ratio	0.130	0.097	0.123	0.117
Body fat (%)	0.169	0.030	0.200	0.010
Body fat (kg)	0.267	0.001	0.289	<0.0001
Visceral fat (kg)	0.264	0.001	0.278	<0.0001
Subcutaneous fat (kg)	0.240	0.002	0.266	0.001
Muscle mass (kg)	0.056	0.476	0.034	0.668
Systolic BP	0.083	0.292	0.030	0.702
Diastolic BP	0.123	0.117	0.090	0.253
FPG	0.157	0.044	0.182	0.020
HbA1c	−0.050	0.597	−0.021	0.825
Total cholesterol	0.064	0.417	0.082	0.296
Triglyceride	0.057	0.467	0.070	0.372
HDL-Cholesterol	−0.017	0.832	−0.016	0.843
LDL-Cholesterol	0.050	0.524	0.065	0.408
CRP	0.031	0.700	0.077	0.347
ALT	−0.073	0.353	−0.075	0.342
Fatty liver	0.122	0.119	0.103	0.189

SUVmean/max, mean/maximum standardized uptake value; TBRmean/max, mean/maximum target-to-background ratio; BP, blood pressure; FPG, fasting plasma glucose; HbA1c, glycated hemoglobin A1c; HDL, high density lipoprotein; LDL, low density lipoprotein; AST/ALT, aspartate/alanine aminotransferase; CRP, C-reactive protein. Data are presented as Pearson’s correlation coefficient (r). p < 0.05 was regarded as statistically significant.

### Independent predictors of vascular inflammation

Univariate linear regression analysis followed by multivariate linear regression analysis was performed to determine the independent predictors of vascular inflammation. For multivariate linear regression analysis, only the total fat among other fat parameters was tested together with other significant parameters because of the multicollinearity. Among the significant parameters identified by univariate linear regression analysis (age, BMI, fasting plasma glucose level, and total fat amount), the total body fat amount was the only significant independent predictor of both TBRmean and TBRmax (TBRmean: regression coefficient = 0.020, p = 0.008; TBRmax: regression coefficient = 0.028, p = 0.005) (Table 6).

**Table 6:** Determinants of carotid TBRmean and TBRmax

	**TBRmean**			**TBRmax**		
	**Regression coefficient**	**SE**	**p-value**	**Regression coefficient**	**SE**	**p-value**
Age	0.003	0.002	0.088	0.003	0.002	0.124
BMI	0.014	0.014	0.339	−0.018	0.019	0.351
FPG	0.001	0.001	0.421	0.002	0.002	0.279
Body fat	0.020	0.007	0.008	0.028	0.010	0.005

TBRmean/max, mean/maximum target-to-background ratio; SE, standard error; BMI, body mass index; FPG, fasting plasma glucose. Data are presented as the regression coefficient and standard error. p < 0.05 was regarded as statistically significant.

### Association of NWO with the highest risk of subclinical vascular inflammation

To assess the risk of subclinical atherosclerosis in the subjects between the NWO and NWL groups, we performed multiple logistic regression analysis using a TBRmax value equal to or greater than the highest quartile as the endpoint. After adjusting for basic clinical factors such as age, fasting plasma glucose level, systolic BP, HDL-cholesterol level, LDL-cholesterol level, and triglyceride level, NWO subjects were 3.1-fold more likely to display subclinical vascular inflammation (OR = 3.071, 95% confidence interval (CI) = 1.292–7.297, p = 0.011). Moreover, the NWO subjects were 2.9-fold more likely to exhibit subclinical vascular inflammation, even after adjusting for age, fasting plasma glucose level, systolic BP, HDL-cholesterol level, LDL-cholesterol level, triglyceride level, and all medication history concerning CVD (OR = 2.887, 95% CI = 1.206–6.914, p = 0.017); no other variables significantly contributed to the vascular inflammation, except for NWO in these models (Table 7).

**Table 7:** Odds ratio for the highest TBRmax (≥ highest quartile TBRmax)

	**NWL**	**NWO**	**p-value**
	**odds ratio (95% CI)**	**odds ratio (95% CI)**	
Model 1	1.00 (reference)	2.384 (1.141–4.982)	0.021
Model 2	1.00 (reference)	3.071 (1.292–7.297)	0.011
Model 3	1.00 (reference)	2.887 (1.206–6.914)	0.017

Model 1, not adjusted; Model 2, adjusted for age, fasting plasma glucose level, systolic BP, HDL-cholesterol level, LDL-cholesterol level, and triglyceride level; Model 3, adjusted for age, fasting plasma glucose level, systolic BP, HDL-cholesterol level, LDL-cholesterol level, triglyceride level, antihypertension drug use, statin use, and antiplatelet agent use. NWL, normal weight lean; NWO, normal weight obesity; CI, confidence interval. Data are presented as odds ratios with the NWL group as a reference (1). p < 0.05 was regarded as statistically significant.

## Discussion

To our knowledge, this is the first study to demonstrate that compared to NWL subjects, NWO subjects have a higher degree of vascular inflammation using ^18^ F-FDG-PET/CT. Furthermore, the findings also reveal the significant contribution of body fat to subclinical vascular inflammation, even in the normal BMI population. Thus, the main finding of the present study is that more active and earlier surveillance of subclinical vascular inflammation may be required to prevent clinical vascular events, even in patients who were not defined as obese by the classical definition.

BMI is calculated as the weight divided by the square of height. Body weight is mainly composed of muscle mass and fat mass. It is well known that muscle is a type of endocrine organ, exercise has an anti-inflammatory effect on vessels, and excess fat mass exerts harmful effects on vascular inflammation [30,31]. Obesity is principally defined as the presence of excess fat mass with elevated cardiometabolic risk. Therefore, although BMI is the most easily used and widely accepted criteria for defining obesity [6], BMI can miscategorize a significant proportion of subjects who have lower muscle mass content and higher body fat levels as those having a same cardiovascular risk as healthy, non-obese subjects [7-10]. Previous studies have indicated the elevation of carotid artery TBR in obese subjects with BMI values of ≥25 kg/m^2^[32] or BMI values of ≥30 kg/m^2^[33]. In addition, several studies identified a persistent positive correlation between TBR and BMI even after adjusting for confounding factors [32,34]. However, none of these studies assessed the possibility of NWO, previously classified as not obese, as a potential risk for vascular inflammation. The importance of our study lies in identifying NWO as a novel risk factor for increased vascular inflammation and in determining that body fat itself, rather than BMI, is more important as a driving force for this subclinical inflammation.

Several reports have indicated that visceral fat may have a greater harmful effect on vascular inflammation than subcutaneous fat [35]. When we analyzed the degree of vascular inflammation in subjects with excess visceral fat or in subjects with excess subcutaneous fat by generating new parameters such as “the ratio of visceral-to-subcutaneous fat amount” or “the ratio of subcutaneous-to-visceral fat amount,” the value of TBRmean and TBRmax were found to be higher in the subjects with higher visceral-to-subcutaneous fat ratio and lower in the subjects with higher subcutaneous-to-visceral fat ratio. This suggests that varying ratios of depot-specific fat amount may have different effects on vascular inflammation (Data not shown). Moreover, we suggest that the ratio of visceral-to-subcutaneous adipose tissue may be essential for vascular inflammation, even in normal weight subjects. However, it should be considered that an increment in the absolute amount of total fat, regardless of whether it involves visceral or subcutaneous fat, may definitely exert a harmful effect on vascular inflammation, even in normal weight subjects, as shown in the present data. Although we could not extensively analyze the depot-specific effect of body fat on vascular inflammation in normal weight subjects in the present study, the findings obtained in the present study indicate that this topic warrants further investigation.

De Lorenzo et al. reported that the levels of inflammatory cytokines interleukin-6 and tumor necrosis factor-α, which are secreted by adipose tissue, are elevated in NWO subjects compared to their NWL counterparts [12]. These fat-derived inflammatory factors can affect the development of atherosclerosis [36,37]. In addition, Yoo et al. suggested that circulating adipocyte fatty acid binding protein, primarily expressed in adipocytes, is an independent determinant of vascular inflammation measured by ^18^ F-FDG-PET/CT [38]. Moreover, the NWO population in the present study also exhibited higher levels of the inflammation marker CRP, although the difference was not significant. Thus, these findings demonstrate that subclinical vascular inflammation may be caused by direct or indirect inflammatory signals from adipose tissue in NWO subjects. NWO subjects may be genetically predisposed to obesity and elevated cardiovascular risk [39], whereas the regulation of caloric excess may prohibit the development of obesity and elevation of cardiovascular risk [40]. As subclinical atherosclerosis can finally be presented as a clinically significant cardiovascular event [41], future strategies to identify susceptible subjects (such as NWO individuals in the present study) and to develop interventions to prohibit the development of a cardiovascular event in the population with subclinical atherosclerosis are warranted. These findings also emphasize that it is critical to identify a useful biomarker for selecting the population at risk for adipose tissue-associated vascular inflammation, including subjects with a normal BMI. The usefulness of the biomarkers may be validated with the non-invasive ^18^ F-FDG-PET/CT imaging technique as conducted in our study.

Before the introduction of ^18^ F-FDG-PET/CT as a non-invasive imaging tool for visualizing the early inflammation of atherosclerosis in the vessel, there was no definite method to detect subclinical atherosclerosis [42,43]. The current methods such as intima-media thickness, coronary computed tomography angiography, and coronary angiography mainly evaluate the structural change of the vessels, which is a manifestation of progressed atherosclerosis [44]. In the present analysis, importantly, some patients in the NWO group had a very high degree of vascular inflammation (TBRmax > 2.0), a finding that has not been observed in NWL subjects thus far. Severe inflammatory lesions may result in a clinically meaningful CVD event [45,46]. In addition, studies involving non-invasive functional imaging of vascular inflammation using ^18^ F-FDG-PET/CT indicated that these highly inflammatory lesions can serve as targets of earlier intervention in NWO subjects, the results of which were promising in a small population [34,47-50]. However, further studies would need to investigate whether the use of this technique results in a clinically significant reduction of future vascular events.

This study has several limitations. Our study used an arbitrary cutoff value of BF% based on the tertiles of BF% to define NWO. However, there is no established cutoff value for delineating the harmful effect of body fat on a cardiometabolic event. Therefore, although arbitrary, we followed the method used in a previously published paper [14]. Determination of the optimal cutoff value of BF% for discriminating the subjects at high risk for future CVD events will be necessary. Second, we used a relatively simple BI method to evaluate the body fat. However, there are concerns that the BI method might underestimate upper body obesity [51], and dual energy X-ray absorptiometry might be more accurate for evaluating BF%. In contrast, the BI method has several advantages such as the avoidance of radiation exposure, simplicity, and low cost, suggesting the utility of BF estimation in large-scale studies [52]. Third, this study is a cross-sectional study that cannot confirm a direct causal relationship between BF and vascular inflammation. Nevertheless, to our knowledge, this is the first study to target the NWO population specifically in a community-oriented cohort. Furthermore, this study has a relatively large sample size among studies on vascular inflammation using ^18^ F-FDG-PET/CT. Nevertheless, a prospective study with a longer study period is warranted in the future to confirm whether vascular inflammation assessed by ^18^ F-FDG-PET/CT is actually associated with CVD events.

## Conclusions

To our knowledge, the present study is the first study to indicate that NWO subjects, who have increased BF% compared to NWL subjects, are associated with a higher degree of vascular inflammation using ^18^ F-FDG-PET/CT, which enables the noninvasive detection of functional/subclinical vascular inflammation. Furthermore, we suggested that BF may be a major contributing factor of vascular inflammation even in normal weight patients. NWO subjects, formerly classified as non-obese with the conventional BMI criteria, may represent a unique subset of patients who should not be excluded from high-risk groups of future CVD events. The findings of the present study warrant future clinical investigations into the uniqueness of NWO patients, particularly regarding the potential for future CVD events.

## Abbreviations

BMI: Body mass index; BF: Body fat; NOW: Normal weight obesity; NWL: Normal weight lean; 18 F-FDG-PET/CT: ^18^ F-fludeoxyglucose positron emission tomography/computed tomography; TBRmean and TBRmax: Mean and maximum target-to-background ratios; NHANES: National Health and Nutrition Examination Survey; KNHANES: Korean National Health and Nutrition Examination Survey; CVD: Cardiovascular disease; BF%: Body fat percentage; BI: Bioelectrical impedance; HbA1c: Glycated hemoglobin A1c; HDL-cholesterol: High-density lipoprotein cholesterol; CRP: C-reactive protein; AST: Aspartate aminotransferase; ALT: Alanine aminotransferase; LDL-cholesterol: Low-density lipoprotein cholesterol; ROI: Region of interest; SUV: Standardized uptake value; SUVmax: Maximal SUV; TBR: Target-to-background ratio; SD: Standard deviation; OR: Odds ratio; CI: Confidence interval.

## Competing interests

The authors declare that they have no competing interests.

## Authors’ contributions

SK designed the study, contributed to acquisition of data, performed statistical analysis, and wrote the manuscript. CK, JSP, and SHK have contributed to the design of study, acquisition of data, and interpretation of data. SPL participated in the design of the study, interpretation of data, and helped to draft the manuscript. MKK and HKK participated in acquisition of data and interpretation of data. KRK contributed to interpretation of data. TJJ contributed to the design of the study, acquisition of data, and extensive interpretation of data. CWA coordinated the study, and contributed to the acquisition and interpretation of data. All authors read and approved the final manuscript.
